# Ibrutinib in Relapsed or Refractory Mantle Cell Lymphoma and Chronic Lymphocytic Leukemia

**DOI:** 10.6004/jadpro.2014.5.5.5

**Published:** 2014-09-01

**Authors:** Derek Peterson, Joanna Schwartz

**Affiliations:** Albany College of Pharmacy and Health Sciences, Colchester, Vermont, and Fletcher Allen Healthcare, Burlington, Vermont

Targeted scientific advancements have produced novel therapies and made significant improvements in the clinical landscape of non-Hodgkin lymphoma (NHL) treatment. Although mantle cell lymphoma (MCL) and chronic lymphocytic leukemia (CLL) are both B-cell lymphocyte subtypes of NHL neoplasms, there are several notable differences.

Mantle cell lymphoma is an aggressive subtype of NHL characterized by a poor prognosis. Approximately 6% of all newly diagnosed NHL patients present with MCL (Pérez-Galán, Dreyling, & Wiestner, 2011). In 2014, a predicted 70,800 American adults will be diagnosed with NHL (Siegel, Ma, Zou, & Jemal, 2014). The clinical course of MCL features a declining survival curve resulting in a median overall survival (OS) of 4 to 6 years (Dreyling &; Hiddemann, 2009).

Chronic lymphocytic leukemia is an indolent leukemia/NHL subtype. It is the most common leukemia in adults. US trends for CLL predict 15,720 new cases and 4,600 deaths among adults in 2014 (Siegel et al., 2014). Chronic lymphocytic leukemia has a variable clinical course due to an assortment of mutations that display resistance to conventional therapy.

Both MCL and CLL are fundamentally caused by a malfunction in the standard B-cell maturation process. However, each is distinct, as the respective disorders occur in separate phases of B-cell differentiation. Mantle cell lymphoma is considered to be a pregerminal center neoplasm resulting from a mutation to naive B cells; CLL is known as a postgerminal center neoplasm developing into a monoclonal expansion of a mutated memory B cell (Jaffe, Harris, Stein, & Isaacson, 2008; Wang et al., 2011).

Currently, there are no curative therapeutic courses for MCL and CLL. Standard frontline therapies for both MCL and CLL consist primarily of chemotherapeutic and chemoimmunotherapeutic agents. Specifically, MCL induction therapy includes a chemotherapeutic backbone along with rituximab (Rituxan) treatment. Current treatment regimens are often followed by stem cell transplants and do demonstrate improved outcomes for MCL patients. Due to the aggressive nature of this type of malignancy, relapse is common and mortality rates are high (Wang et al., 2013).

Conventional CLL treatment consists of combinations. often involving fludarabine, cyclophosphamide, and rituximab. Treatment options for relapsed disease have been associated with increased toxicity and often become a concern in elderly populations (Byrd, Jones, Woyach, Johnson, & Flynn, 2014; National Comprehensive Cancer Network [NCCN], 2014). Established CLL treatment regimens have also been associated with poor response due to chromosomal mutations, including del17p and del11q.

A novel mechanism of targeted therapies for CLL and MCL has been investigated in an effort to provide valuable treatment options for these complicated populations, by improving efficacy as well as offering less toxic treatment options for elderly patients. A first-in-class Bruton’s tyrosine kinase (BTK) inhibitor, ibrutinib (Imbruvica) has demonstrated efficacy in clinical trials for relapsed and refractory MCL and CLL (Byrd et al., 2013; Byrd et al., 2014; Wang et al., 2013). This article will prepare the oncology advanced practitioner (AP) for the management of patients receiving ibrutinib by reviewing all relevant clinical evidence.

Bruton’s tyrosine kinase functions as an essential terminal kinase of the B-cell receptor (BCR) signaling cascade required for normal B-cell development (Akinleye, Chen, Mukhi, Song, & Liu, 2013). It was initially identified in X-linked agammaglobulinemia (XLA), a rare immune deficiency disorder (Akinleye et al., 2013) mediated by a loss-of-function BTK mutation, resulting in the absence of B cells (Wiestner, 2013). The role of BTK in B-cell proliferation and survival provides a pharmaceutical target for mature B-cell lymphoproliferative disorders.

## Pharmacology and Mechanism of Action

As previously mentioned, ibrutinib is a first-in-class small-molecule inhibitor of BTK, which has been identified as an essential kinase enzyme in the BCR signaling cascade. The BCR functions as the receptor for antigen; upon activation, it recruits numerous kinases and adaptor proteins for signal propagation. B-cell receptor signaling subsequently leads to the activation of various downstream effectors responsible for B-cell life cycle management (Woyach, Johnson, & Byrd, 2012). Ultimately, the transcription factors activated through BCR signaling play prominent roles in promoting cell growth, proliferation, and survival of normal and malignant B cells. Recent evidence suggests that antigen-dependent activation may play a part in the pathogenesis and disease progression of both MCL and CLL (Wiestner, 2013).

Ibrutinib exerts its inhibitory functions on BTK through various mechanisms. It primarily acts as an irreversible allosteric inhibitor of BTK’s enzymatic activity by selectively binding to a cysteine residue within inhibitory segments. Ibrutinib has shown the ability to activate apoptosis as well as disrupt prosurvival pathways, proliferation, and mobilization of B cells (Akinleye et al., 2013; Pharmacyclics, Inc., 2014). The targeted effects of ibrutinib provide a promising contrast to the off-target tissue effects of cytotoxic therapies.

Ibrutinib is hepatically metabolized into several metabolites, primarily by CYP3A and to a lesser extent CYP2D6. The half-life of ibrutinib ranges from 4 to 6 hours, with about 80% of the dose excreted in the feces. Bruton’s tyrosine kinase active site occupancy in peripheral blood mononuclear cells of > 90% has been demonstrated up to 24 hours after its administration (Pharmacyclics, Inc., 2014).

## Clinical Trials

Ibrutinib was granted US Food and Drug Administration (FDA) approval for relapsed or refractory MCL and CLL through phase II clinical studies. As part of the accelerated approval process, additional phase II and III trials are in progress to confirm its efficacy and safety.

**Mantle Cell Lymphoma**

A phase II, multicenter, open-label, single-agent trial of ibrutinib (560 mg once daily) in 111 patients with relapsed or refractory MCL displayed durable efficacy throughout the study. Patients were differentiated into 2 groups: those who previously received at least 2 cycles of bortezomib (Velcade) therapy and those who had received fewer than 2 complete cycles of bortezomib therapy. The primary endpoint was overall response rate (ORR), described as either a partial response or a complete response according to the Revised International Working Group Criteria for NHL. Secondary endpoints evaluated included response duration, progression-free survival (PFS), OS, and safety.

A median follow-up of 15.3 months revealed 46 patients had continued receiving treatment, whereas 65 patients had discontinued treatment (disease progression accounted for 50 discontinuations). The ORR for all patients was 68%, with 21% of patients exhibiting a complete response. Response to ibrutinib was not affected by baseline risk factors or previous treatment failure. In patients who presented with lymph node measurements ≥ 5 cm in diameter (n = 43), 63% displayed a response to treatment. In patients who showed a response (n = 75), a median response duration of 17.5 months was observed (95% confidence interval [CI] = 15.8 mo–not reached). Time to response was evaluated at a median of 1.9 months, with a median time to complete response of 5.5 months. The median PFS among all patients was 13.9 months (95% CI = 7.0 mo–not reached). Finally, at study completion, a median OS was not reached; however, a 58% OS rate was observed at 18 months (Wang et al., 2013).

**Chronic Lymphocytic Leukemia**

A phase Ib/II multicenter, open-label, single-agent trial of ibrutinib (420 or 840 mg once daily) was conducted in 85 men and women diagnosed with relapsed or refractory CLL. This trial demonstrated a high ORR (71%), with long durations of response (26-month estimated rate of PFS and OS: 75% and 83%, respectively) for the entire treatment population (Byrd et al., 2013).

A phase III multicenter, open-label, head-to-head, single-agent, randomized controlled trial of ibrutinib and ofatumumab (Arzerra) in patients with relapsed or refractory CLL was conducted in 391 patients. Enrolled patients were randomized to receive either ibrutinib or ofatumumab per the following protocol: oral ibrutinib 420 mg once daily until disease progression or intolerable toxic drug effects, IV ofatumumab for up to 24 weeks at a starting dose of 300 mg at week 1, then 2,000 mg weekly for 7 weeks, and then every 4 weeks for 16 weeks. An amendment was made to the protocol 4 months after the final patient randomization, allowing of atumumab patients whose disease had progressed to receive ibrutinib. The duration of PFS was the primary endpoint evaluated, along with secondary endpoints of OS and response rate.

A median follow-up of 9.4 months revealed that ibrutinib significantly prolonged PFS, as a median was not yet reached for this cohort. The ofatumumab arm exhibited a median PFS of 8.1 months. Statistical analysis using hazard ratios was conducted to demonstrate the efficacy of ibrutinib. The hazard ratio for disease progression or death in the ibrutinib arm was 0.22 (95% CI = 0.15–0.32;* p* < .001). An evaluation of each treatment arm at 6 months revealed 88% of ibrutinib patients were alive with no disease progression compared with 65% of ofatumumab patients.

An important dataset from this study verified the results seen in phase II data regarding ibrutinib’s efficacy in patients with cytogenic abnormalities. The hazard ratio for disease progression or death of patients with a 17p13.1 deletion was 0.25 (95% CI = 0.14–0.45). Six months into the study, 83% of del17p patients receiving ibrutinib were alive with no disease progression compared with 49% of del17p ofatumumab patients. Independent analysis of response rates displayed a significantly higher rate of 63% partial responses (20% with lymphocytosis) in ibrutinib patients and 4% in ofatumumab patients (odds ratio, 17.4; 95% CI = 8.1–37.3; *p* < .001; Byrd et al., 2014).

## Adverse Events

Lymphocytosis, traditionally used as a marker for disease progression, is common in patients receiving ibrutinib treatment, as it causes the movement of lymphocytes from lymph nodes into the peripheral blood. This elevation in lymphocyte count parallels reduction in lymph node size and is not an indicator of disease progression. The clinical studies described here revealed a median peak lymphocyte count at 4 weeks followed by a transient decrease to normal levels (Byrd et al., 2013; Wang et al., 2013).

Evidence supports the fact that ibrutinib is a well-tolerated agent, even in elderly and comorbid populations, as seen in clinical trials. Adverse events reported are characterized largely as grade 1 or 2; see Table 1 (Byrd et al., 2014; Byrd et al., 2013; Wang et al., 2013).

**Table 1:**
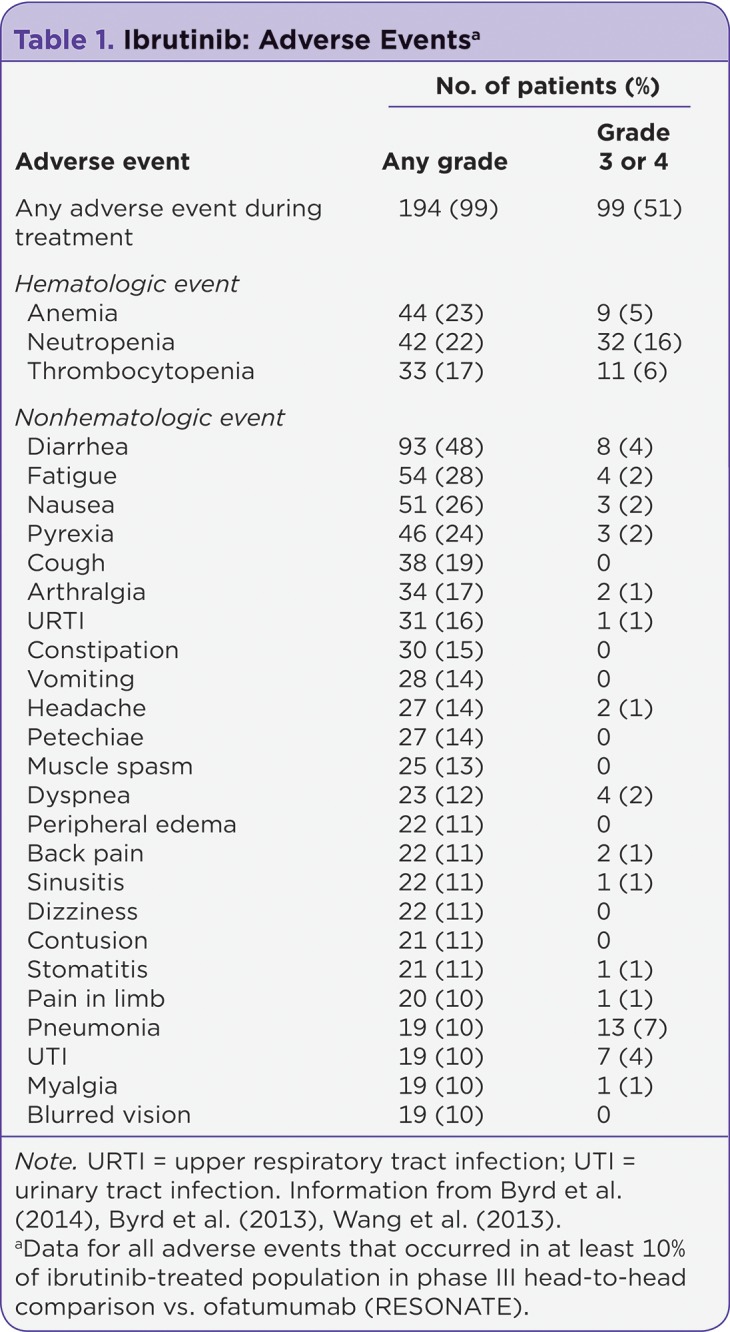
Ibrutinib: Adverse Events

## Current Place in Therapy

Clinical studies as well as the FDA approval status of ibrutinib support its use as a treatment option for MCL and CLL, specifically for patients with relapsed or refractory disease who have received at least one prior therapy. The favorable response rates and safety profile demonstrated by single-agent ibrutinib treatment provide evidence for its use in patients who may not tolerate or respond to conventional second-line options. The efficacy of targeting BTK in patients with a poor prognosis (CLL: del17p and del11q genomic mutations) has created further interest in targeting other essential protein kinases of the BCR signaling cascade.

Several protein kinase inhibitors (PKIs), which inhibit BCR signaling, are currently in phase II and III of development. The efficacy of ibrutinib observed in phase II and III data as well as early proof-of-concept studies of other PKIs might suggest a shift in the treatment paradigm for B-lymphocyte malignancies in the future. In addition to their notable efficacy, PKIs are associated with fewer nontarget toxicities than other second-line MCL and CLL therapies. Concerns have been raised about the long-term ramifications of inhibiting BCR signaling, but there are limited data to support any definite conclusions (Jahangiri, Friedberg, & Barr, 2014).

Current National Comprehensive Cancer Network guidelines suggest the use of ibrutinib in CLL/small lymphocytic lymphoma as a preferred second-line agent in patients of all ages (safety < 18 years not evaluated) following a short response with first-line therapy regardless of prognosis. In stage I/II MCL, ibrutinib is reserved as an option for patients having received induction therapy, and upon disease progression or relapse, having also received chemotherapy. In stage II bulky/III/IV MCL, ibrutinib is recommended in several scenarios: as a treatment option for patients demonstrating a complete response to induction therapy but relapsing after consolidation treatment or as a treatment option after either disease progression or a partial response to induction therapy (NCCN, 2014).

## Implications for the Advanced Practitioner

Ibrutinib offers a convenient once-daily oral treatment option for MCL and CLL patients who have progressed to refractory stages of disease. Due to the existing unmet medical needs and its substantial efficacy in B-cell malignancies, ibrutinib is likely to be prescribed frequently.

The recommended daily dose of ibrutinib differs per indication: 560 mg (four 140-mg capsules) for use in MCL patients and 420 mg (three 140-mg capsules) for use in CLL patients. Both regimens should be taken by mouth once daily at a consistent time. The capsules should be administered without respect to meals and with a full glass of water. For a missed dose, the patient should take the capsules as soon as possible on the same day and resume the normal administration time the following day (Pharmacyclics, Inc., 2014).

Ibrutinib is extensively metabolized via the CYP3A pathway; therefore, both strong and moderate CYP3A inhibitors and inducers may markedly affect its drug concentrations. Concurrent use of strong CYP3A inhibitors should be avoided if possible. However, the labeling information does note that in the event a strong CYP3A inhibitor is used for ≥ 7 days, interrupting ibrutinib treatment for the duration of the CYP3A inhibitor may be considered. The dose of ibrutinib with concurrent moderate CYP3A inhibitors must be reduced to 140 mg (MCL and CLL), which includes not only some commonly used agents such as diltiazem, verapamil, ciprofloxacin, clarithromycin, and fluconazole, but also grapefruit, grapefruit juice, and even the Seville oranges found in marmalade. Conversely, as strong CYP3A inducers can decrease serum ibrutinib levels approximately 10-fold, concurrent use should be avoided (Pharmacyclics, Inc., 2014).

Hepatic metabolism and drug excretion in the feces account for 80% of the drug, whereas less than 10% of the drug is accounted for in urine. Therefore, drug levels are not expected to be significantly affected by renal impairment. Ibrutinib should be avoided with baseline significant hepatic impairment, although to date, it has not been studied in patients with severe renal or hepatic impairment (Pharmacyclics, Inc., 2014).

Adverse events with ibrutinib observed in clinical trials were primarily mild (grades 1 or 2) and not a common cause of treatment discontinuation. The most common side effects leading to treatment discontinuation, amounting to 4% to 6.5% in the studies discussed, were either due to infection or bleeding events. Bleeding events of grade 3 or higher have occurred in approximately 5% of patients on ibrutinib, whereas events of any grade (including bruising) are much more common, with 50% to 60% of patients on ibrutinib reporting incidents. Therefore, caution is advised in patients using concomitant antiplatelet or anticoagulant therapies.

It may be appropriate to interrupt therapy with ibrutinib for 3 to 7 days before and after surgery upon determination of the risks vs. the benefits. Respiratory tract infections have proved to be the most common infections, with pneumonia cases of grade 3 or higher occurring in 7% of the phase III trial population published. Grade 3 or higher cytopenias, including thrombocytopenia, neutropenia, and anemia, have been observed in 30% of trial populations. Due to the increased risk of infection and cytopenias, myelosuppression should be assessed monthly, with a complete blood cell count; APs should counsel patients to report any fevers or symptoms. As treatment-emergent elevations in serum creatinine have been reported, it is recommended that APs advise patients about proper hydration as well as monitor creatinine levels every 3 months. Additionally, reducing risk and/or managing other toxicities such as infection and diarrhea; maintaining proper hand hygiene; using loperamide; and staying on a low-fiber diet should be included in patient education. Dose modifications are recommended in the instance of severe toxicity, which includes low complete blood cell counts; see Table 2 (Pharmacyclics, Inc., 2014).

**Table 2:**
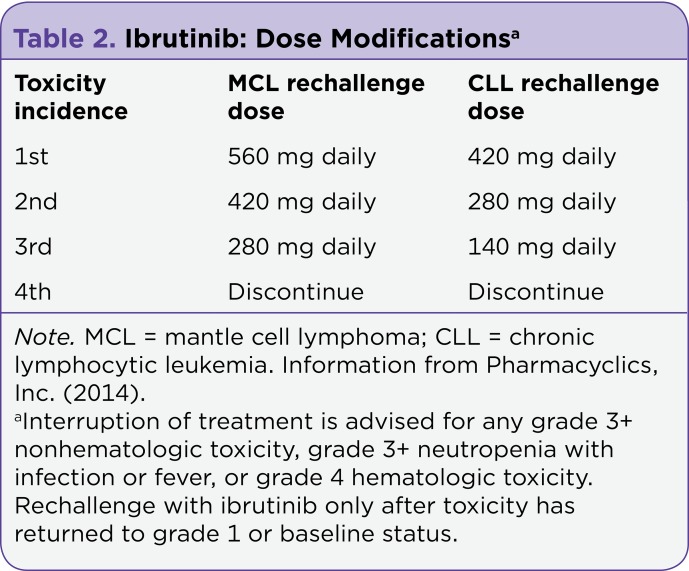
Ibrutinib: Dose Modifications

Lymphocytosis is commonly seen in patients receiving ibrutinib and is not an indication of progressive disease. Peak lymphocyte counts are seen at a median of approximately 4 weeks, with a return to baseline at a median of 8 total weeks (Byrd et al., 2014; Pharmacyclics, Inc., 2014).

An ibrutinib dose of 140 mg daily is recommended for patients receiving concomitant moderate CYP3A inhibitors. Although ibrutinib is supplied in only 90- and 120-count bottles, the FDA has recently approved repackaging to provide smaller amounts at the time of dispensing. This change allows patients to receive a reduced dose to obtain a 1-month supply (Pharmacyclics, Inc., 2014; Institute for Safe Medication Practices, 2014). Patient access assistance is available through the manufacturing companies, as a significant cost may be realized with continued therapy (Pharmacyclics, Inc., 2014; Truven Health Analytics, 2014).

In study populations, there have been a small number of patients who have relapsed during ibrutinib therapy. Recent investigations into these patients have provided a baseline understanding of the mechanisms of resistance involved. A cysteine-to-serine mutation in the BTK binding site of ibrutinib, as well as additional mutations directly downstream to phospholipase Cã2, has been characterized, the latter of which were determined to be gain-of-function mutations leading to autonomous BCR activity (Woyach et al., 2014).

## Summary

The introduction of targeted agents that inhibit the BCR signaling pathway is likely to change the treatment paradigm for B-cell malignancies. The efficacy and safety demonstrated by the first-in-class BTK inhibitor ibrutinib in both MCL and CLL have provided a single-agent option for patients with refractory or relapsed disease. In a keystone phase III trial, ibrutinib displayed superior PFS, OS, and ORR to ofatumumab in patients with relapsed CLL. Responses demonstrated with ibrutinib have been robust and observed regardless of standard determinants of poor prognosis, such as purine analog resistance and cytogenic abnormalities.

Ibrutinib as a single agent has already garnered excitement in the oncology community because of its durable responses and increased tolerability compared with previous therapies. Moving forward, treatment applications of the novel BTK inhibitor may expand after the completion of ongoing clinical studies. The safety and efficacy of ibrutinib in additional malignancies, subsets of currently indicated populations, and in combination with current frontline regimens are currently being evaluated. Expect ibrutinib along with other PKIs in development to continue challenging standard therapies for an integral role in B-cell malignancy management.
